# Genome-Wide Identification of NAC Transcription Factors and Their Functional Prediction of Abiotic Stress Response in Peanut

**DOI:** 10.3389/fgene.2021.630292

**Published:** 2021-03-09

**Authors:** Pengxiang Li, Zhenying Peng, Pingli Xu, Guiying Tang, Changle Ma, Jieqiong Zhu, Lei Shan, Shubo Wan

**Affiliations:** ^1^Bio-Tech Research Center, Shandong Academy of Agricultural Sciences, Shandong Provincial Key Laboratory of Crop Genetic Improvement, Ecology and Physiology, Jinan, China; ^2^College of Life Science, Shandong Normal University, Jinan, China

**Keywords:** peanut, NAC transcription factor, gene family, abiotic stress, expression analysis

## Abstract

The NAC transcription factor (TF) is one of the most significant TFs in plants and is widely involved in plant growth, development, and responses to biotic and abiotic stresses. To date, there are no systematic studies on the *NAC* family in peanuts. Herein, 132 AhNACs were identified from the genome of cultivated peanut, and they were classified into eight subgroups (I–VIII) based on phylogenetic relationships with *Arabidopsis* NAC proteins and their conserved motifs. These genes were unevenly scattered on all 20 chromosomes, among which 116 pairs of fragment duplication events and 1 pair of tandem duplications existed. Transcriptome analysis showed that many *AhNAC* genes responded to drought and abscisic acid (ABA) stresses, especially most of the members in groups IV, VII, and VIII, which were expressed at larger differential levels under polyethylene glycol (PEG) and/or ABA treatment in roots or leaves. Furthermore, 20 of them selected in response to PEG and ABA treatment were evaluated by quantitative real-time polymerase chain reaction. The results showed that these genes significantly responded to drought and ABA in roots and/or leaves. This study was helpful for guiding the functional characterization and improvement of drought-resistant germplasms in peanuts.

## Introduction

The NAC transcription factor (TF) is one of the most abundant types of TFs in plants; its designated name comes from the homologous proteins encoded by *NAM*, *ATAF1/ATAF2*, and *CUC2* (Souer et al., 1996). NAC TFs have similar N-terminal structures (Aida et al., 1997), which consist of five subdomains (A–E), and usually contain approximately 160 amino acid residues. Among them, subdomains A, C, and D are highly conserved. The nuclear localization signal that involves the recognition process of the NAC TF localizes to the C and D subdomains. Although the E subdomain has lower conservation, it may participate in developmental regulation and cooperation with the D domain in a tissue-specific manner. The C-terminus of the NAC protein contains a transcriptional activation region, which has higher diversity, and some repeated amino acids, such as serine, threonine, and proline, are enriched in this region (Tran et al., 2010; Wang and Dane, 2013; Kim et al., 2016; Mathew and Agarwal, 2018).

In angiosperms, the NAC TF family has large numbers of members, for example, 117 NAC members in *Arabidopsis* (Ooka et al., 2003), 151 in rice (Ooka et al., 2003), 152 in soybean (Le et al., 2011) and corn (Shiriga et al., 2014), and 110 members in potato (Singh et al., 2013). NAC proteins regulate multiple biological processes in plants, including seed and embryo development (Duval et al., 2002; Sperotto et al., 2009), shoot tip meristem formation (Kim S.G. et al., 2007), fiber development (Ko et al., 2007), leaf senescence (Guo and Gan, 2006), cell division (Kim et al., 2006), and lateral root growth (Xie et al., 2000). Additionally, many *NAC* genes participate in the response to abiotic stresses in plants, such as drought, salinity, cold, and water logging (Hu et al., 2006; Jeong et al., 2010; Nuruzzaman et al., 2012). In *Arabidopsis*, overexpressing the *NAC* genes *ANAC019*, *ANAC055*, and *ANAC072* enhanced tolerance to drought in transgenic lines (Tran et al., 2004). Overexpression of the *AtNAC2* gene improved the salt tolerance of transgenic plants (He et al., 2005). The NAC TF gene *TaNAC2* from wheat also responded to drought, salt, cold, and abscisic acid (ABA) stress. Overexpressing *TaNAC2* in *Arabidopsis* improved their tolerance to these stresses (Mao et al., 2012). Overexpression of the *SNAC1*, *OsNAC6*, *OsNAC5*, *OsNAC45*, and *OsNAC63* genes in rice can significantly increase drought and salt tolerance (Hu et al., 2006; Nakashima et al., 2007; Song et al., 2011; Trapnell et al., 2012). *GmNAC11* and *GmNAC20* in soybean are involved in the response to low temperature, and their overexpression can enhance the low-temperature tolerance of transgenic soybean plants (Hao et al., 2011).

Cultivated peanut (*Arachis hypogaea*) is a major oilseed crop and cash crop widely cultivated worldwide; its yield per unit area is higher than those of other oilseed crops. In China, peanut is always planted in arid and semiarid hilly regions because of its higher tolerance to drought and barren conditions. The growth period of peanuts is exceptionally susceptible to drought stress, which affects the yield and quality of peanuts (Yan et al., 2007). Recently, the functions of several peanut *NAC* genes in abiotic stress tolerance were investigated. Overexpression of the *AhNAC2* (renamed *AhNAC53* in our article) gene in *Arabidopsis* significantly improved drought and salt resistance (Liu et al., 2011, 2014). Overexpression of *AhNAC3* (renamed *AhNAC12* in our article) in tobacco increased the drought resistance of transgenic tobacco (Liu et al., 2013). Transgenic tobacco plants with *AhNAC4* (renamed *AhNAC53* in our article) transcripts enhanced their drought resistance by regulating stomatal opening and closing, reducing transpiration, and improving water use efficiency (Tang et al., 2017). However, to date, there has been no systematic study about the NAC family in peanut. In this article, 132 *NAC* genes were identified from the genome of cultivated peanut and were classified into eight main groups according to their phylogenetic relationships. Additionally, their gene structure, chromosome location, and conserved motifs were analyzed using bioinformatics methods. Furthermore, the function of these *AhNAC* genes in responding to drought stress was predicted by a genome-wide survey, and their expression patterns after treatment with drought and ABA were analyzed by quantitative real-time polymerase chain reaction (qRT-PCR). This study will help elucidate the functions of these *NAC* genes in the drought response and in the molecular breeding of drought resistance in peanut.

## Materials and Methods

### Identification of NAC TFs in Peanut

The peanut protein sequences were downloaded from the peanut library^1^. The HMM file of the NAM domain (PF02365) was retrieved from the Pfam database^2^ and was used to search the NAC family proteins with an *e* value less than 0.001 in the peanut protein database by HMMER 3.0 local software. Subsequently, some incomplete and redundant amino acid sequences were deleted, and the possible AhNACs were confirmed by BLASTP and CDD programs. The number of amino acids, molecular weight (MW), and theoretical isoelectric point of each NAC protein sequence were calculated using ExPASy^3^.

### Sequence Alignment and Phylogenetic Analysis of AhNAC TFs

*Arabidopsis* NAC protein sequences were downloaded from the *Arabidopsis* Information Resource^4^, and sequence alignment of NAC TFs in peanut and *Arabidopsis* was performed by the MUSCLE method (Kumar et al., 2016). Subsequently, an unrooted phylogenetic tree was established by MEGA 7 using the neighbor-joining (NJ) method with a p-distance model and 1,000 bootstrap repeats (Kumar et al., 2018).

### Chromosome Location and Gene Duplication of AhNAC Genes

The position of each *AhNAC* gene and the sizes of every peanut chromosome were extracted from the gff3 annotation files in the peanut library, and the chromosome distribution of genes was visualized using MapChart software (Voorrips, 2002). The duplication analysis of *AhNAC* genes considered the following two aspects: (1) the length of the shorter aligned sequence covered more than 70% of the longer sequence; and (2) the identity between two aligned sequences was greater than 70% (Gu et al., 2002; Yang et al., 2008). *AhNAC* genes were aligned with the whole peanut genome by local BLAST software, and gene duplications were then identified by the MCScanX (Wang et al., 2012) algorithm with the default setting (*e* ≤ 1e-10). A gene duplication diagram was drawn using Circos software (Krzywinski et al., 2009).

### Gene Structure and Conserved Motif Analyses

The motif analysis tool MEME^5^ was used to analyze the conserved motifs of the NAC family according to any number of repetitions and parameters with a maximum motif number of 50. The gene structures were assessed with the Gene Structure Display Server^6^. The MEME theme was further annotated with InterPro^7^. The transmembrane (TM) domain was analyzed with TMHMM Server v. 2.0^8^

### Promoter Analysis of NAC Family Genes

The 2,000-base-pair (bp) sequences upstream of the start codon ATG of *AhNAC* genes were retrieved from the peanut genome using TB tools software (Chen et al., 2020) and were submitted to the online software PlantCARE^9^ for the analysis of *cis*-acting elements. The draft of the element distribution on each promoter was sketched using TBtools.

### Expression Profiles Under Different Stresses Using RNA-Seq Data

The Illumina RNA-seq data (PRJNA553073) derived from roots and leaves of the peanut variety Fenghua No. 1 (FH1) were downloaded from the Sequence Read Archive^10^. The seedlings of FH1 used for sequencing were cultivated in 1/2 MS_0_ medium at room temperature for 10 days and were then treated with 20% polyethylene glycol (PEG) 6000 or 20 mg/L ABA for 24 h. RNA-seq data were analyzed by Hisat2, Samtools, and Cufflinks to obtain FPKM values. A heat map showing tissue-specific expression profiles (log_2_FPKM values) was made using TBtools.

### RNA Extraction and qRT-PCR Analysis

The FH1 seedlings were cultivated in nutrient soil at room temperature for 10 days and irrigated twice daily using Hoagland solution during seedling growth. Then, the seedlings were treated with Hoagland solution supplemented with 20% PEG6000 or 20 mg/L ABA, and their leaves and roots after treatment for 0 (control, CK) and 24 h were picked and stored at −80°C. Total RNA was extracted using a Quick RNA Isolation Kit 3.0 (Huayueyang Biotechnology, Beijing, China) according to the manufacturer’s protocol and was then digested with RNase-free DNase I (TaKaRa, Dalian, Liaoning Sheng, China). The quality and quantity of RNA were determined by agarose gel electrophoresis and ultraviolet spectrophotometry (BioPhotometer Plus, Eppendorf, Germany). First-strand cDNA was synthesized by the PrimeScript II 1st Strand cDNA Synthesis Kit (TaKaRa, Dalian, Liaoning Sheng, China).

The relative expression levels of *AhNAC* genes under drought and ABA stress conditions were analyzed by qRT-PCR using the *ACTIN7* gene as an internal control and were calculated using the 2^–ΔΔCt^ method. Primer Premier 5.0 software was used to design qRT-PCR primers, and PCR was performed with the following steps: first, predenaturing at 95°C for 10 min, then 40 cycles of 95°C for 15 s and 60°C for 1 min, and finally 95°C for 30 s and 60°C for 15 s. The experiments were performed in three biological replicates, with technical triplicates per biological repeat. All primer sequences are shown in Supplementary Table 1.

## Results

### Identification of the AhNAC Gene Family

A total of 196 NAC sequences were retrieved after searching the peanut protein database using the NAM conserved domain (Pfam: PF02365) by the local HMMER 3.0 software. Among them, some sequences with incomplete NAM domains or with *e* values greater than 1*e*^–3^ were excluded by BLASTP and CDD verification. Ultimately, 132 NAC TFs in peanut with the canonical NAM domain were identified and named *AhNAC1* to *AhNAC132* according to their physical locations on the chromosome.

The *AhNAC* genes vary in length from 535 bp (*AhNAC93*) to 9,732 bp (*AhNAC53*), and their coding proteins range from 127 (AhNAC1) to 740 (AhNAC122) amino acids. The MWs of AhNACs were between 15.2 kDa (AhNAC1) and 83.19 kDa (AhNAC122), with the majority of them ranging from 20 to 50 kDa in peanut. Their average MW was 40.08 kDa. The predicted *pI* ranged from 4.67 (AhNAC123) to 9.77 (AhNAC56), with an average of 6.85, among which 60 AhNACs had *pI* > 7, and 72 AhNACs had *pI* < 7 (Supplementary Table 2).

### Phylogenetic Analysis and Classification of NAC Genes

**FIGURE 1:**
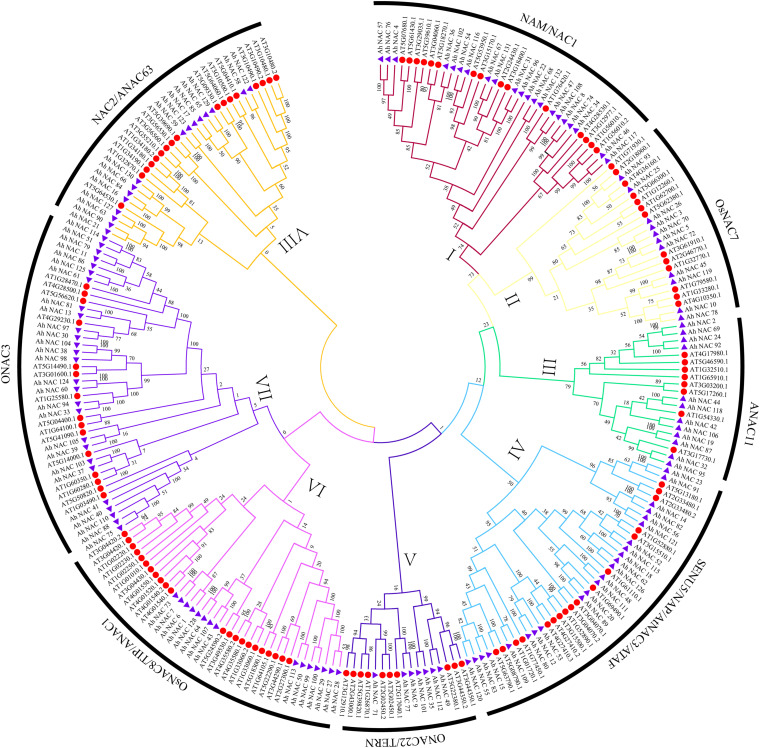
An unrooted phylogenetic tree representing the relationships among the NAC proteins of peanut and *Arabidopsis*. All full-length protein sequences were aligned by MUSCLE, and the tree was generated using the neighbor-joining (NJ) method in MEGA7 software. The I–VIII subgroups are represented by different colors, the purple triangles represent NAC proteins from peanut, and the red circles indicate the *Arabidopsis* protein.

To explore the evolutionary relationship between AhNAC TFs, an unrooted phylogenetic tree was constructed using 132 AhNAC proteins in peanut and 117 AtNACs from *Arabidopsis* (Supplementary Table 3). According to the clustering result of NACs from *Arabidopsis thaliana* and rice in the article by Ooka et al. (2003), all members of NAC TF mentioned previously were divided into eight groups, the NAM/NAC1 (group I), OsNAC7 (group II), ANAC11 (group III), SENU5/NAP/AtNAC3/ATAF (group IV), ONAC22/TERN (group V), OsNAC8/TIP/ANAC1(group VI), ONAC3(group VII), and NAC2/ANAC63 (group VIII) groups (Ooka et al., 2003); the largest group (group VII) consists of 42 proteins, including 27 peanut NACs, and the smallest (group V) has 17 proteins, including seven NAC members of peanut (Figure 1). The other groups from most to least had 40 (23 AhNACs, group IV), 37 (15 AhNACs, group VI), 36 (21 AhNACs, group I), 33 (16 AhNACs, group VIII), 24 (11 AhNACs, group II), and 20 (12 AhNACs, group III) members.

Previous studies on NAC TFs indicated that genes clustered in one subgroup might have similar functions (Tran et al., 2009; Nuruzzaman et al., 2010; Le et al., 2011; Ahmad et al., 2018; Liu et al., 2019). It is worth noting that a variety of representative abiotic stress–related genes appeared in group IV. For example, the expression of the *Arabidopsis RD26* gene (AT4G27410.2) was induced by drought-related high salinity (Fujita et al., 2004). Overexpression of *ATAF1* (AT1G01720.1) in *Arabidopsis* increased plant sensitivity to ABA, salt, and oxidative stresses (Wu et al., 2009). Our results found that there were 23 AhNAC members in group IV, of which 17 *Arabidopsis* NACs were divided into three subclades. AhNAC53 clustered together with *Arabidopsis* AT1G52890.1, AT3G15800.1, and AT4G27410.2, and their sequence similarities were 57.82%, 69.37%, and 76.79%, respectively. AhNAC12 and AhNAC80 also belonged to this subclade and had higher homology with AhNAC53, and their sequence identities were 60% and 60.36%, respectively. AhNAC80 had 60.98% sequence similarity with *Arabidopsis* ANAC55 (AT3G15500.1) in the same group. Overexpression of *AtNAC2* (AT3G15510.1) in *Arabidopsis* can improve resistance to drought by stimulating secondary root development under drought conditions (He et al., 2005). AhNAC62, AhNAC126, AhNAC18, AhNAC115, AhNAC52, AT1G52880.1, and ATNAC2 were clustered into the same subclade. Among them, AhNAC62 and 126 are orthologous genes from the A and B genomes and have 85.50% and 71.20% sequence similarity with ATNAC2, respectively. Some NACs with a TM motif 1-like (NIL) of *Arabidopsis*, including AT1G33060 (NTL2), AT3G44290 (NTL4), AT3G49530 (NTL6), AT2G27300 (NTL8), AT4G35580 (NTL9), AT1G01010 (NTL10), AT4G01540 (NTL12), and AT4G01550 (NTL13), were clustered in group VI. Peanut AhNACs (1, 6, 7, 27, 28, 29, 43, 50, 64, 73, 99, 100, 107, 113, and 128) gathered in this group, and AhNAC64, 128, and AhNAC107 had higher sequence similarity with NTL2 and NTL9, respectively. In group VIII, the homologous genes AhNAC127 and AhNAC63 from the A and B genomes were clustered in the same branch as *Arabidopsis* XND1 (AT5G64530.1), and both proteins had 70.16% sequence similarity with XND1. XND1 could indirectly affect aquaporin function to reduce its tolerance to drought stress (Tang et al., 2018). The other members in this subclade, AhNAC21 and AhNAC90, also had 65.1% and 65.63% sequence identity with XND1 (Supplementary Table 4).

### Chromosomal Mapping and Duplication Analysis of the Peanut NAC Gene

**FIGURE 2:**
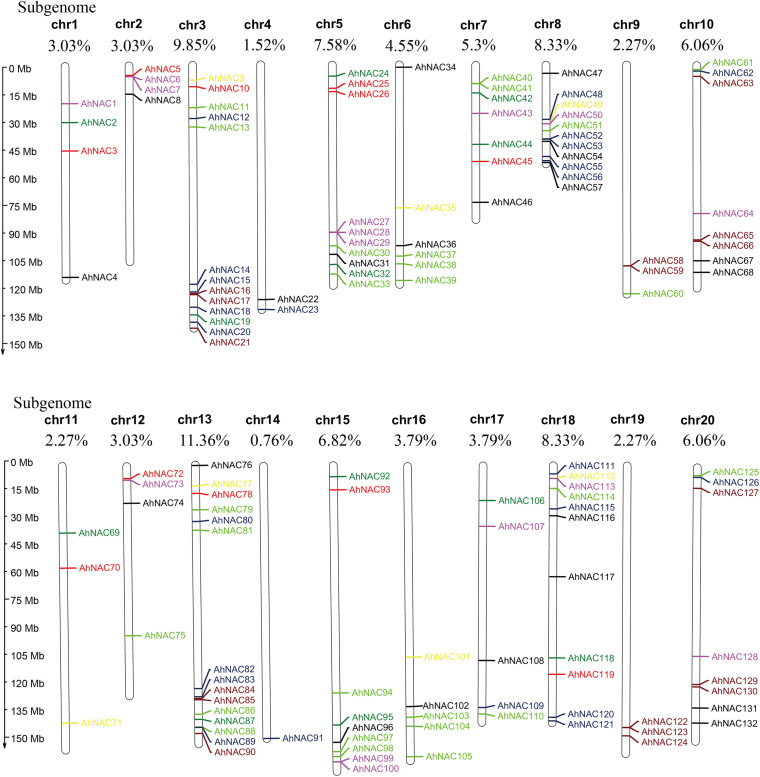
Chromosomal physical map showing the uneven distribution of *AhNAC* genes on each chromosome. Different colors indicate the different subgroups. The chromosome length is shown to the left of the map, and the serial numbers of chromosomes (chr1–chr20) and the distribution frequencies are shown on the top of each chromosome. Black represents group I, red represents group II, green represents group III, blue–black represents group IV, yellow represents group V, purple represents group VI, light green represents group VII, and brown represents group VIII.

To confirm the chromosome distribution of *AhNAC* genes, a physical map was constructed with MapChart. It appeared that 132 *NAC* genes were unevenly mapped on all 20 chromosomes, half of which were derived from the A genome (Chr 1–10) and the other half from the B genome (Chr 11–20, Figure 2). Most genes were present on chromosomes 3 and 13 belonging to the A and B genomes, respectively, accounting for 9.85% (13 genes) and 11.36% (15 genes) of the total gene numbers, and the fewest genes were scattered on chromosome 4 from the A genome and chromosome 14 of the B genome, accounting for 1.52% (2 genes) and 0.76% (1 gene), respectively. Some corresponding chromosomes from the A/B genome, such as chromosomes 2 and 12 (4 genes), 8 and 18 (11 genes), 9 and 19 (3 genes), and 10 and 20 (8 genes), had equal numbers of genes (Figure 2).

**FIGURE 3:**
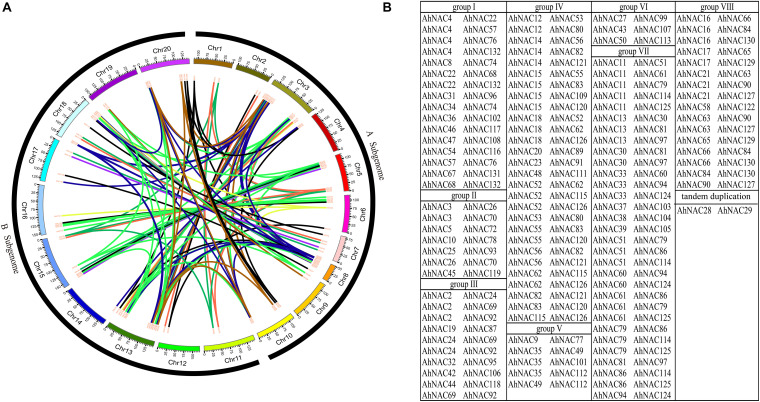
Collinearity analysis results indicating that a number of duplication events existed in *AhNAC* genes. **(A)** The colinear relationship among *AhNAC* genes is shown as a circle map. The red line indicates the duplication events between pairs, and the different-colored bars represent the different chromosomes. **(B)** The statistics of duplicated gene pairs according to their assigned groups. Black represents group I, red represents group II, green represents group III, blue–black represents group IV, yellow represents group V, purple represents group VI, light green represents group VII, and brown represents group VIII.

A collinearity analysis by MCScanX software found that among 132 *AhNAC*s, 116 pairs of segmental duplication genes were identified; these were divided into eight groups according to the phylogenetic tree (Figure 3). Maximum segmental duplication events occurred in group VII (31 pairs), followed by group IV (28 pairs), whereas groups V and VI (5 pairs and 3 pairs) showed far fewer segmental duplication events. Only one pair of tandem duplication genes, *AhNAC28* and *AhNAC29*, was detected in group VI (Figure 3B).

### Conserved Motifs and Gene Structure of AhNAC

**FIGURE 4:**
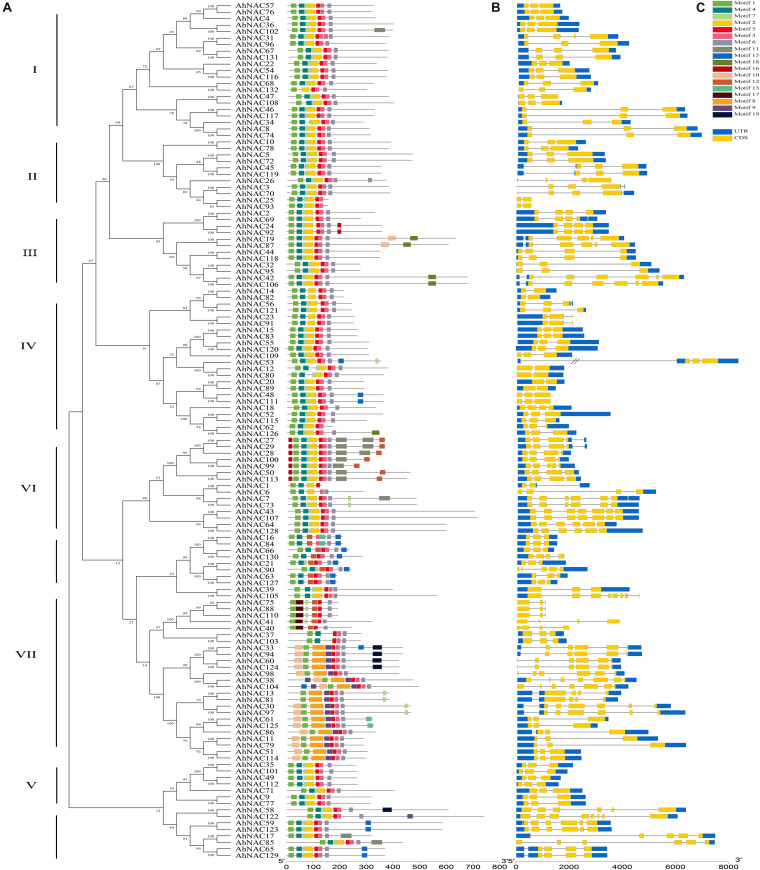
Analysis of the conserved motifs of AhNAC family members and their coding gene structures. **(A)** The phylogenetic tree of peanut AhNACs was constructed in MEGA7 using the NJ method. **(B)** Schematic diagrams of putative conserved motifs of 132 AhNAC proteins. The conserved motifs were found using the MEME tool and are shown by different colored boxes. **(C)** Sketch map of the exon–intron structures of 132 *AhNAC* genes. The blue and yellow boxes indicate the regions of the 5′- and 3′-UTRs and the exons, respectively, and the black lines indicate the introns in genes.

To further investigate the features of AhNACs, conserved motifs were verified by searching the MEME database, and homologous relationships among their coding proteins were constructed according to the motif composition. A total of 20 motifs were identified in all AhNAC members. The AhNAC gene family was classified into eight groups (I–VIII), which was similar to the unrooted phylogenetic tree of NAC proteins (Figure 4A). Group VIII was further divided into two subgroups, A and B, because of a lack of motifs 7 or 12 at the N-terminus and different lengths and motifs at the C-terminus. Almost every AhNAC had motifs 1, 3, 5, and 6, which were considered the NAC domains through InterProScan searching. Most of the AhNAC proteins also contained motifs 2, 4, and 7, which included all members of groups I–VI and group VIIIB and two members of group VII. Sequence analysis found that motifs 1 and 4, 2 and 7, 3 and 5, and 6 corresponded to subdomains A to E localized at the N-terminus of NAC proteins (Supplementary Table 5) (Diao et al., 2018; Liu et al., 2019). The majority of AhNACs in group VII also had motifs 8, 9, and 10, and almost half of the AhNACs in group VI had motif 16 at the N-terminus. The C-termini of AhNACs were less conserved, even though in the same group, they had different motif compositions. There were almost no new motifs found at the C-termini of groups I, II, and V members. Motifs 10, 16, and 18 appeared in some members of group III, and motif 15 appeared in three members of group IV. Nearly half of the members in group VI contained motifs 11, 12, and 16, and four members in group VII had motif 19 at their C-termini (Figure 4B). Analysis of the TM domains of the AhNACs at the C-terminus revealed that AhNAC7, 43, 50, 64, 73, 107, 113, and 128 of group VI all had one TM domain, and AhNAC19, 42, 87, and 106 in group III and AhNAC59 and 123 in group VIII contained two TM domains, whereas the other members had no TM structures at the C-terminus (Supplementary Table 6). Most closely related members of the phylogenetic tree showed a characteristic motif with the same arrangement and location, suggesting that NAC members clustered in the same subgroup may have similar biological functions.

A sketch of the exon–intron structure of each *AhNAC* gene is displayed in Figure 4C. The numbers and lengths of exons in most *AhNAC* genes were essentially in accord with the classification of the phylogenetic relationship of the AhNAC family. In groups I and V, apart from *AhNAC31*, *AhNAC67*, and *AhNAC131*, the other genes had three exons. There were two to four exons in groups II and IV, but the genes in different groups had largely distinct sizes and arrays of introns, especially *AhNAC53*, which contained a large intron in its 5’ UTR. In groups III and VII, most *AhNAC* genes had three to four exons, and the others had six to seven exons, whereas these genes displayed diverse exon–intron structures. In group VI, the exon numbers of *AhNAC* genes were different from 3 to 7. The gene structures between groups VIIIA and VIIIB showed many variant patterns; most genes in VIIIA had three exons and relatively shorter introns, whereas the VIIIB genes had more than five exons and some longer introns. These results indicated the diversity of the *AhNAC* gene structure (Figure 4C).

### Expression Patterns of AhNAC Genes Treated With Different Stresses

**FIGURE 5:**
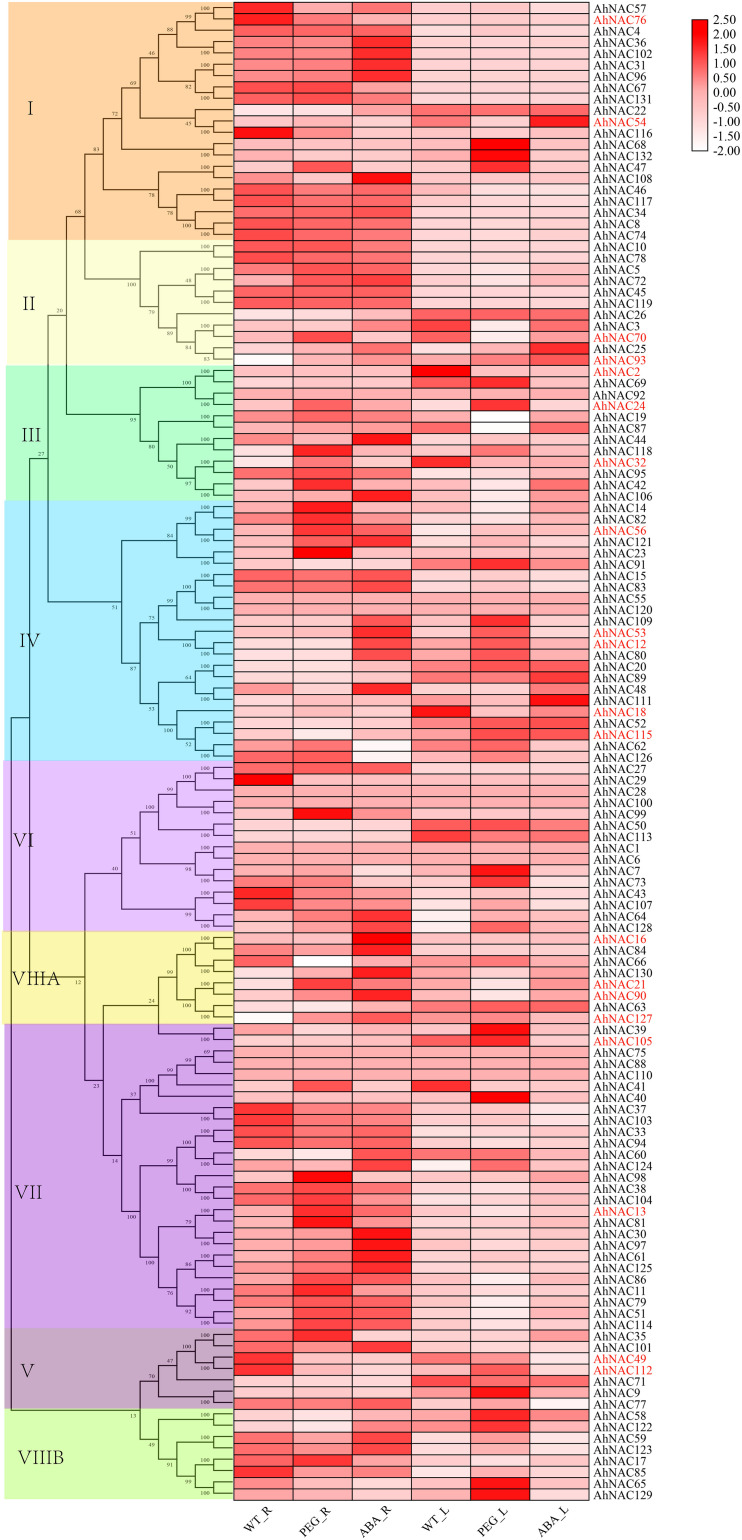
Heat map presenting the expression patterns of *AhNAC* genes in roots and leaves treated with PEG and ABA. The relative expression values were transformed by log2 of FPKM values and are displayed as colored boxes from light pink to red, and genes selected by qPCR are indicated in red font.

In order to discover the roles of the *AhNAC* genes, the expression profiles were summarized by analyzing the RNA-Seq data (PRJNA553073) of the roots and leaves under PEG and ABA treatment, and heat maps of the relative expression levels were established using TBtools. The results showed that in either roots or leaves, the expression levels of most genes in groups I and II did not change significantly under the ABA and PEG treatments. However, *AhNAC47*, *AhNAC68*, and *AhNAC132* of group I in leaves were upregulated under PEG treatment, whereas *AhNAC54* was downregulated by PEG and induced by ABA; in roots, the expression levels of six genes (*AhNAC22*, *31*, *36*, *96*, *102*, and *108*) of group I were improved by the treatment of ABA, whereas only *AhNAC47* was induced by PEG, and *AhNAC54*, *57*, *76*, and *108* were downregulated. In group II, *AhNAC3* and its homologous gene *AhNAC70* had different response patterns to treatment in roots and leaves; their expression was reduced in leaves treated with PEG, whereas in roots, *AhNAC3* and *AhNAC70* were induced by ABA and PEG, respectively; the expression levels of *AhNAC25* and *93* were enhanced by treatment with both PEG and ABA (Figure 5, Supplementary Table 7).

In leaves, most of the genes in groups VI, VII, and VIII were less affected by PEG and ABA treatment, but *AhNAC7*, *64*, *73*, and *128* in group VI; *AhNAC39*, *40*, *105*, and *124* in group VII; and *AhNAC58*, *65*, *122*, and *129* in group VIIIB were all significantly upregulated by PEG treatment. Few members in groups III and V responded to ABA, and six genes (*AhNAC2*, *19*, *32*, and *87* downregulated, and *AhNAC92* and *118* expressed in the opposite manner) of group III and two genes (*AhNAC9* and *112*) of group V were affected by PEG. In group IV, most genes responded to PEG treatment; for example, *AhNAC12*, *53*, *62*, *80*, *91*, and *109* were upregulated by PEG treatment; only three genes (*AhNAC48*, *89*, and *111*) were upregulated under ABA treatment; and *AhNAC20*, *52*, and *115* significantly responded to both conditions (Figure 5, Supplementary Table 7).

In roots, the subclade members with higher homology in group IV, including *AhNAC14*, *23*, *56*, *82*, and *121*, were more responsive to PEG treatment, and only *AhNAC56* and *121* were upregulated under ABA treatment, whereas the higher homologous *AhNAC*s (*AhNAC12*, *53*, *80*, and *109*) in another subclade and *AhNAC48* were markedly upregulated under ABA treatment. Many genes in group VII also responded to PEG and ABA treatment; for example, six genes (*AhNAC13*, *51*, *61*, *86, 114 a*nd *125*) were upregulated under PEG and ABA treatment, and *AhNAC37* and *AhNAC103* showed the opposite trend; another six genes (*AhNAC11*, *38*, *41*, *81*, *98*, and *104*) were induced only by PEG treatment, and four genes (*AhNAC30*, *60*, *97*, and *124*) were upregulated by ABA treatment. Almost all of the genes in group VIIIA responded to ABA and PEG, among which *AhNAC21*, *90*, *127*, and *AhNAC130* were upregulated, and only *AhNAC66* was downregulated; the expression levels of *AhNAC16* and *84* were improved under ABA treatment. In the remaining groups, the minority of genes were affected by PEG and ABA. *AhNAC32*, *42*, *92*, and *118* in group III; *AhNAC35* in group V; *AhNAC99* in group VI; and *AhNAC17* in group VIIIB showed upregulated trends under PEG treatment, and *AhNAC44* and *106* in group III, *AhNAC101* in group V, *AhNAC64* and *128* in group VI, and *AhNAC59*, *122*, and *123* in group VIIIB were upregulated under ABA treatment. The genes showing decreased expression under PEG and ABA treatment included *AhNAC49* and *112* in group V, *AhNAC29*, *43*, and *107* in group VI and *AhNAC85* in group VIIIB (Figure 5, Supplementary Table 7).

**FIGURE 6:**
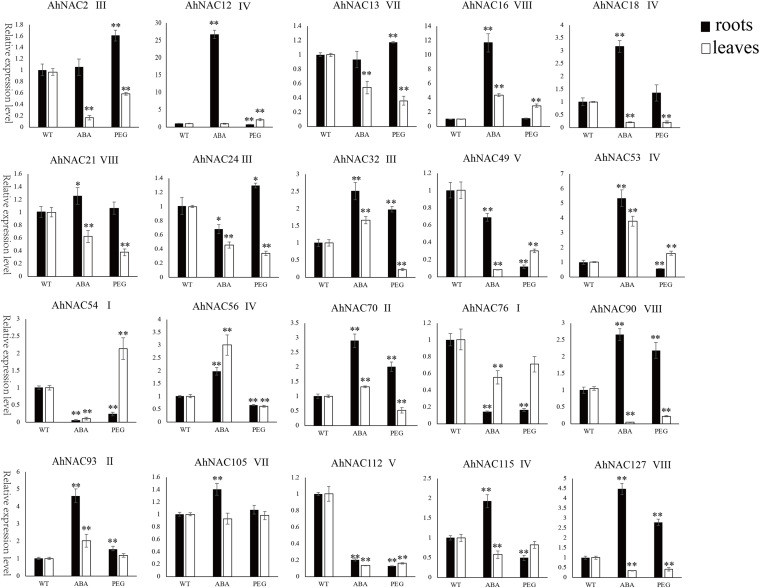
The expression patterns of 20 *AhNAC* genes in roots and leaves after PEG and ABA treatment were verified by qRT-PCR. The relative expression levels of AhNAC genes in untreated peanut (WT) and PEG and ABA treated for 24 h were compared. Data were calculated from three biological replicates, and the significance of variants between the control and treatment groups was analyzed by one-way ANOVA. Significant differences at the levels of ^∗^*p* < 0.05 and ^∗∗^*p* < 0.01.

To verify the expression profiles of some *AhNAC*s that responded to PEG and ABA treatment, 20 *AhNAC* genes, of which *AhNAC54* and *76* were from group I; *AhNAC70* and *93* were from group II; *AhNAC2*, *21*, and *56* were from group III; *AhNAC12*, *18*, *53*, *56*, and *115* were from group IV; *AhNAC49* and *112* were from group V; *AhNAC13*, *105* were from group VII; and *AhNAC16*, *21*, *90*, and *127* were from group VIII, were selected for qRT-PCR analysis. The results showed that the majority of genes analyzed by the two methods had similar expression patterns in response to ABA and PGE treatment, especially in roots. However, some genes without obvious responses to ABA and/or PEG in the RNA-seq data showed inconsistent patterns when analyzed by qRT-PCR. For example, the qRT-PCR results showed that *AhNAC13*, *21*, *76*, *90*, and *127* were downregulated, and *AhNAC16* was upregulated significantly by both ABA and PEG treatment, and the expression of *AhNAC53* was induced by not only PEG but also ABA in leaves; in roots, *AhNAC18* was markedly upregulated under both ABA and PEG treatment. The response patterns of *AhNAC56* and *115* to ABA and PEG in roots and leaves found by qRT-PCR were inconsistent with the RNA-seq data. The qRT-PCR results showed that the expression level of *AhNAC56* significantly increased under ABA treatment and decreased with PEG treatment in both roots and leaves, and *AhNAC115* was only markedly induced by ABA in roots and was repressed by ABA in leaves and by PEG in the two organs. It was inferred that the samples from different picking times used for detection might lead to discrepancies in the results (Figure 6, Supplementary Table 8).

## Discussion

NAC TF is one of the largest plant-specific transcriptional regulators that play critical roles in stress responses and various developmental processes. Previous studies have identified NAC families in many plant species. However, there is still no detailed information available on the NAC family in peanut. Here, we identified 132 *AhNAC* genes from the peanut genome, which were classified into eight groups in accordance with the clusters of *Arabidopsis* AtNACs. The average MW of AhNACs (40.08 kDa) falls in the MW range (40–55 kDa) of the NAC proteins from 160 plant species (Mohanta et al., 2020). In addition, 116 pairs of segmental duplications and one pair of tandem duplications occurred, resulting in the large *AhNAC* gene family in the peanut genome. Mohanta et al. reported more AhNAC members (162) and larger duplicated events (161) in the genome of *A. hypogaea*. Similarly, there were multiple family members derived from a great quantity of duplicated events in some species of dicots and monocots (Ooka et al., 2003; Mohanta et al., 2020), and more duplication events probably occurred in these species after differentiation from their earliest ancestors. Several studies have indicated that the NAC family undergoes two discontinuous expansion processes during species evolution: the first occurred in land plants diverged from other streptophytes, whereas the second took place in flowering plants after their divergence from other vascular plants (Zhu et al., 2012; Maugarny-Calès et al., 2016; Mohanta et al., 2020). Their establishment in early diverged land plants was conserved during subsequent plant evolution, although many gene duplications and losses occurred (Zhu et al., 2012). Gene duplications are considered to be one of the primary driving forces in the evolution of genomes and gene families and are the foundation of producing new genes and new functions (Bowers et al., 2003; Moore and Purugganan, 2003). In peanut, these duplicated genes were observed in all groups, with more duplicated events in groups VII and IV. Multiple members of these two groups participated in responding to abiotic stresses. Domestication of *A. hypogaea* took place ≈4,500 years ago, and the modern cultivated tetraploid improved many agronomic traits but lost some resistance traits of wild relatives (Yin et al., 2019). This finding is probably due to the selective pressure conferred by environmental cues to facilitate the growth and development of plants. The peanut living environment is changeable and affected by the environment. The expansion of the NAC TF family might be beneficial for adaptability to the environment during peanut domestication.

Most of the NAC proteins that have been studied to date are involved in the responses to abiotic and biotic stress and the regulation of developmental processes (Pei et al., 2013; Kim S.G. et al., 2007; Kim et al., 2008; Li et al., 2016; Shahnejat-Bushehri et al., 2016). Under adverse environments, several NAC members of group IV in *Arabidopsis*, such as *ANAC019*, *ANAC055*, *ANAC072 (RD26)*, and *ATAF1* (*ANAC002*), participate in the stress response (Fujita et al., 2004; Wu et al., 2009). *Arabidopsis RD26* is involved in the response to drought and high salinity in an ABA-dependent manner (Fujita et al., 2004). ANAC019 acts as an upstream regulator to induce the expression of several key drought-induced genes, including *DREB2A*, *DREB2B*, *ARF2*, *MYB21*, and *MYB24*, synergistically promoting both drought response and flower development (Sukiran et al., 2019). Overexpression of *ATAF1* in *Arabidopsis* increased plant sensitivity to ABA, salt, and oxidative stresses (Wu et al., 2009). Recent studies have shown that overexpression of the *ATAF1* gene of *Arabidopsis* enhances the transcription of the stress-related genes *OsLEA3*, *OsSalT1*, and *OsPM1* and improves salt tolerance in rice (Liu et al., 2016). In this study, many *AhNAC* genes, especially the members of group IV, responded to ABA treatment and/or PEG treatment, implying that they were associated with regulating the stress response and ABA signaling. For instance, peanut *AhNAC109* was significantly induced in roots by ABA, which has 68.39% sequence similarity to ATAF1 from *Arabidopsis*. Shao et al. found that *AhNAC1* (renamed *AhNAC109* in this study) played a role in seed development and response to abiotic stress (Shao et al., 2008; Wu et al., 2009). *AhNAC53* (known as *AhNAC2* in the study of Liu et al.) has 50% identity with *RD26*, which had lower water losses under drought stress and higher chlorophyll contents under salt stress in transgenic leaves, and its overexpression lines in *Arabidopsis* were more sensitive to ABA in root growth, seed emergence, and stomatal closure than those in the wild-type *Arabidopsis* (Liu et al., 2011, 2014; Tang et al., 2017). *AhNAC12* (known as *AhNAC3* in the study of Liu et al.) was verified to have higher expression under ABA treatment in roots and was significantly upregulated under PEG treatment in leaves. *AhNAC12* and *AhNAC80*, as well as *AhNAC53*, were clustered in the same subclades, and their sequence similarities reached 60% and 60.36%, respectively. A previous study confirmed that overexpressing *AhNAC3* (i.e., *AhNAC12*) in tobacco could activate the *SOD*, *P5SC*, *LEA*, and *ERD10C* genes and enhance the drought tolerance of transgenic tobacco (Liu et al., 2013). Taken together, it was speculated that these genes, which are evolutionarily close, might play similar functions in abiotic stress responses; however, their regulatory roles and mechanisms in response to stress need to be explored in subsequent research.

Some NIL genes were found to respond to different abiotic stresses differentially in plants. In *Arabidopsis*, 13 NAC members have α-helix TM motifs in the C-terminal region, and most of them are upregulated under stress conditions, suggesting their possible involvement in stress responses (Kim S.Y. et al., 2007; Wang and Dane, 2013). The expression levels of *NTL1* and *NTL11* and *NTL4* and *NTL7* were upregulated specifically by heat (37°C) and cold (4°C), respectively, and *NTL6* expression was dramatically induced by NaCl. Notably, some *NTL*s, such as *NTL2* and *NTL3*, were broadly influenced by cold, drought, and NaCl (Kim S.G. et al., 2007). In rice, eight genes clustered in the TIP subgroup showed upregulation by abiotic stress, among which three genes (*Os01g15640*, *Os06g01230*, and *Os08g06140*) share high similarity with *Arabidopsis NTL6* (Nuruzzaman et al., 2010). *Os02g57650*, another TIP subgroup gene, was highly induced in the roots and leaves of rice under severe drought stress (Nuruzzaman et al., 2012). In our analysis of phylogenetic relationships, eight AhNACs (AhNAC7, 43, 50, and 64 and their orthologous AhNAC73, 107, 113, and 128) with one TM motif at the C-terminus, along with major *Arabidopsis* NTLs, were clustered in the OsNAC8/TIP/ANAC1 group (group VI) (Supplementary Table 6). Our RNA-Seq analysis also revealed that six of those genes were affected by PEG and/or ABA in roots and/or leaves. *AhNAC64* and *128* were induced by PEG and ABA in both roots and leaves, and *AhNAC7* and *73* were upregulated only in leaves under PEG treatment, whereas *AhNAC43* and *107* were downregulated in roots treated with both PEG and ABA. These six genes were found to be increased at higher levels in PEG-treated roots than in untreated roots in the RNA-Seq data (accession no. SRP093341) released by Zhao et al. (2018). Notably, the response patterns to PEG of *AhNAC43* and *107* and *AhNAC7* and *73* were obviously inconsistent with our analysis (Supplementary Table 9). This might be attributed to the difference in sampling time under drought conditions. Root samples in the study of Zhao et al. were collected at 0, 6, 12, 18, 24, and 48 h poststress and were pooled for RNA-Seq, whereas in our study, sampling times before 24 h and at 48 h were absent. Some NAC genes respond rapidly to abiotic stress and increase their expression to high levels. For instance, the expression of *ONAC095* in rice leaves was significantly and rapidly upregulated within 3 and 6 h by dehydration and ABA, showing 2.2- to 3.9- and 3.3-fold increases, respectively (Huang et al., 2016). *AtNTL6* was expressed within 10 min after cold treatment and continued to increase, reaching a peak at 18 h (Seo et al., 2010). Therefore, the expression profiles of the *AhNACs* involved in the shock response to drought and ABA might be missed in the current study.

Roots are important organs for the uptake of water and nutrients. The ability of plants to adapt to rhizosphere drought stress requires the reprogramming of root growth and development (Lee et al., 2017). Recent studies have shown that improvement of the root system, such as longer roots and larger root diameters, can improve crop yield under water-limited conditions (Ghanem et al., 2011; Meister et al., 2014; Rogers and Benfey, 2015). Several NAC TFs have also been shown to promote root growth in some crop species and *Arabidopsis*. Overexpression of *AtNAC2* in transgenic *Arabidopsis* plants resulted in the promotion of lateral root development (He et al., 2005). *GmNAC20* and *GmNAC4* also have roles in promoting lateral root formation in soybean (Hao et al., 2011; Quach et al., 2014). *OsNAC5* transgenic rice could upregulate *GLP*, *PDX*, *MERI5*, and *O-methyltransferase* genes related to root growth and development, resulting in larger roots and thicker root diameters and higher yields under drought conditions (Jeong et al., 2013). Rice *OsNAC6* also mediates root structural adaptations for drought tolerance (Lee et al., 2017). Overexpressing *OsNAC9* altered its root architecture, involving an enlarged stele and aerenchyma, which enhanced the drought-resistance phenotype (Redillas et al., 2012). Peanuts *AhNAC62* and *126* had higher homology with *AtNAC2*, and their expression levels in roots were significantly induced by PEG; *AhNAC53* and *109*, markedly induced by ABA in roots, had higher sequence similarity with *OsNAC5* and *OsNAC6*, *GmNAC20*, *GmNAC4* and *OsNAC9.* Whether they had similar functions in regulating root development under drought conditions or some functional redundancy needs to be further investigated.

## Conclusion

In our study, a comprehensive *in silico* analysis including evolutionary, chromosomal location, gene structure, and regulatory elements of the *NAC* gene family in peanut was performed. A total of 132 *AhNAC* genes were identified and classified into eight groups. Their differential expression patterns under PEG and ABA treatment were analyzed. The results showed that many *AhNAC* genes were involved in the response to ABA and/or PEG in roots and leaves, especially the majority of genes in groups IV, VII, and VIII. These findings could be helpful for characterizing the genes’ functions and guiding the breeding of novel drought-resistant germplasms in peanuts.

## Data Availability Statement

The datasets presented in this study can be found in online repositories. The names of the repository/repositories and accession number(s) can be found in the article/Supplementary Material.

## Author Contributions

LS designed and conceived the research. PL and LS wrote the manuscript. PL, ZP, and CM analyzed the data. PL, PX, GT, and JZ performed the experiments. LS and SW revised the manuscript. All authors read and approved the manuscript.

## Conflict of Interest

The authors declare that the research was conducted in the absence of any commercial or financial relationships that could be construed as a potential conflict of interest.

## Footnotes

1 https://www.peanutbase.org/search/gene/

2 http://pfam.xfam.org/

3 https://www.expasy.org/

4 http://www.arabidopsis.org/

5 http://meme-suite.org/

6 http://gsds.cbi.pku.edu.cn/

7 http://www.ebi.ac.uk/interpro/

8 http://www.cbs.dtu.dk/services/TMHMM/

9 http://bioinformatics.psb.ugent.be/webtools/plantcare/

10 https://www.ncbi.nlm.nih.gov/sra/
